# Alzheimer’s Biomarkers are Correlated with Brain Connectivity in Older Adults Differentially during Resting and Task States

**DOI:** 10.3389/fnagi.2016.00015

**Published:** 2016-02-08

**Authors:** Yang Jiang, Haiqing Huang, Erin Abner, Lucas S. Broster, Gregory A. Jicha, Frederick A. Schmitt, Richard Kryscio, Anders Andersen, David Powell, Linda Van Eldik, Brian T. Gold, Peter T. Nelson, Charles Smith, Mingzhou Ding

**Affiliations:** ^1^Department of Behavioral Science, University of Kentucky College of Medicine, Lexington, KY, USA; ^2^Sanders-Brown Center on Aging, University of Kentucky, Lexington, KY, USA; ^3^The Magnetic Resonance Imaging and Spectroscopy Center, University of Kentucky, Lexington, KY, USA; ^4^J Crayton Pruitt Family Department of Biomedical Engineering, University of Florida, Gainesville, FL, USA; ^5^Department of Epidemiology, University of Kentucky College of Public Health, Lexington, KY, USA; ^6^Department of Neurology, University of Kentucky College of Medicine, Lexington, KY, USA; ^7^Department of Biostatistics, University of Kentucky College of Public Health, Lexington, KY, USA; ^8^Department of Anatomy and Neurobiology, University of Kentucky College of Medicine, Lexington, KY, USA

**Keywords:** CSF biomarkers, Aβ_42_ peptides, pTau_181_, default-mode network, short-term memory task, global cognitive status, predictors of AD

## Abstract

β-amyloid (Aβ) plaques and tau-related neurodegeneration are pathologic hallmarks of Alzheimer’s disease (AD). The utility of AD biomarkers, including those measured in cerebrospinal fluid (CSF), in predicting future AD risk and cognitive decline is still being refined. Here, we explored potential relationships between functional connectivity (FC) patterns within the default-mode network (DMN), age, CSF biomarkers (Aβ_42_ and pTau_181_), and cognitive status in older adults. Multiple measures of FC were explored, including a novel time series-based measure [total interdependence (TI)]. In our sample of 27 cognitively normal older adults, no significant associations were found between levels of Aβ_42_ or pTau_181_ and cognitive scores or regional brain volumes. However, we observed several novel relationships between these biomarkers and measures of FC in DMN during both resting-state and a short-term memory task. First, increased connectivity between bilateral anterior middle temporal gyri was associated with higher levels of CSF Aβ_42_ and Aβ_42_/pTau_181_ ratio (reflecting lower AD risk) during both rest and task. Second, increased bilateral parietal connectivity during the short-term memory task, but not during rest, was associated with higher levels of CSF pTau_181_ (reflecting higher AD risk). Third, increased connectivity between left middle temporal and left parietal cortices during the active task was associated with decreased global cognitive status but not CSF biomarkers. Lastly, we found that our new TI method was more sensitive to the *CSF* Aβ_42_-connectivity relationship whereas the traditional cross-correlation method was more sensitive to levels of CSF pTau_181_ and cognitive status. With further refinement, resting-state connectivity and task-driven connectivity measures hold promise as non-invasive neuroimaging markers of Aβ and pTau burden in cognitively normal older adults.

## Introduction

Amyloid plaques and neurofibrillary tangles are the defining pathological features of Alzheimer’s disease (AD). The accumulation of β-amyloid (Aβ) peptide in the brain is considered to indicate preclinical AD in non-demented individuals (Morris et al., 2006). In animal models, Aβ disrupts neural activity at the synaptic level and at network circuits with other brain regions (Palop and Mucke, 2010). In humans, about 20–50% of cognitively normal older adults have Aβ deposits imaged with positron emission tomography using Pittsburgh compound B (PIB; Sperling et al., 2009; Quigley et al., 2011).

Although neurofibrillary tangles (tau pathology) and tau-related neurodegeneration are considered the next stage of the AD progression (Jack et al., 2013), cortical Aβ-negative individuals can also have neurodegeneration (as tracked by tau biomarkers; Dickerson and Wolk, 2012; Crary et al., 2014; Kovacs et al., 2016). Furthermore, cognitive functioning in normal older adults is reported to be associated with tau pathology but not with Aβ pathology (Wirth et al., 2013). A recent review on the correlation of AD neuropathological changes with cognitive impairment suggests that the severity of cognitive impairment correlates best with the burden of neocortical neurofibrillary tangles (Nelson et al., 2012). Furthermore, the amount of tau protein in human cerebrospinal fluid (CSF), independently of Aβ, is a relatively strong predictor of progression of AD or cognitive decline (Jagust, 2013; Kandimalla et al., 2013; Wirth et al., 2013; Koch et al., 2014).

In cognitively normal older adults, the use of Aβ and tau biomarkers as predictors of functional brain response patterns is still being optimized. The current study investigated the relationship between CSF biomarkers Aβ_42_ or phosphorylated Tau_181_ (pTau), and functional connectivity (FC) during resting or during a short-term memory task in cognitively normal older adults. Evidence from functional neuroimaging has revealed decreased brain activity during cognitive tasks, compared to that during resting state, in a group of brain regions that include the posterior precuneus/posterior cingulate cortex (PCC; Brodmann area, BA 31/7), medial prefrontal cortex (mPFC), lateral parietal, and inferior temporal gyrus (BA 20), collectively known as the default-mode network (DMN; Shulman et al., 1997; Raichle et al., 2001). In cognitively normal older adults, stronger deactivations in DMN were found to be associated with better memory performance (Parasuraman and Jiang, 2012). By contrast, AD patients’ poor memory performance has been linked to a failure to deactivate the DMN deactivation (Greicius et al., 2004).

Amyloid plaques form within regions of the DMN and the pathology is associated with functional activation differences (Buckner and Vincent, 2007). There are unknown complexities in the relationship between network connectivity during resting state (Biswal et al., 1995) and during task performance, and AD biomarkers (Kandimalla et al., 2011), age, and cognitive status in older persons. Our study investigated whether non-invasive neuroimaging indicators that can predict future AD risk in cognitively intact older adults. Short-term memory (e.g., working memory) undergoes significant early declines in aging and AD dementia (Grady et al., 2001). A recent study demonstrated that working memory task-induced deactivation in the DMN can be predicted by resting-state glutamate and GABA concentrations in the PCC/precuneus (Hu et al., 2012). To test the hypothesis that FC among DMN component regions during resting state and task performance are differentially associated with CSF-based levels of AD biomarkers (Aβ and tau) and cognitive status, we analyzed both resting-state and task-driven fMRI data in cognitively normal, well-characterized aged volunteers. For task activation, we developed an older-adult friendly version of a short-term memory task that is sensitive to DMN activity. We also applied a novel time series-based measure of FC called total interdependence (TI) in addition to the traditional cross-correlation (CC) analysis. Based on the literature and our previous work, we predict that resting and task states detect differential default network alterations due to subclinical AD pathology indexed by CSF biomarkers.

## Materials and Methods

### Participants

Participants in the current study were healthy, community dwelling, older adults (Mean age 77 years old) enrolled in a longitudinal study of aging and brain health at the University of Kentucky Alzheimer’s Disease Center (UK-ADC). These individuals undergo annual neuropsychological testing, clinical assessment, and blood sample collection that are detailed elsewhere (Schmitt et al., 2012). In addition to these procedures, each participant also completes The National Alzheimer’s Coordinating Center’s Uniform Data Set (Version 2.0; Morris et al., 2006) protocol. Twenty-seven cognitively normal older adults (14 females) participated in the current neuroimaging study. The CSF samples were obtained via lumbar puncture (see below) on the same day that neuroimaging was completed. All research activities were approved by the University of Kentucky Institutional Review Board, and all participants provided written informed consent.

### Cognitive and Clinical Assessments

All participants completed the standard UDS neuropsychological battery (version 1.0) (Weintraub et al., 2009), including the mini-mental state exam (MMSE), within 18 months of the MRI scanning. We computed the UDS *T*-score, a standardized summary score that measures global cognition, for each participant as described in Mathews et al. (2014). The *T*-score has a mean of 50 and a SD of 10. A consensus team that included the examining neurologist, neuropsychologist, and psychometrist made the determination of normal cognition based on the results of the UDS assessment.

### CSF Collection and Analysis

Study neurologists collected fasting lumbar CSF samples from participants using a 20-gage needle and 15-mL sterile polypropylene collection tubes. Samples were stored in single-use 0.5 ml aliquots in a −80°C freezer. The CSF biomarkers Aβ_42_ (pg/ml) and pTau_181_ (pg/ml) were analyzed in the laboratory of Mary Jo LaDu at the University of Illinois at Chicago using INNOTEST ELISA kits by Innogenetics, Gent, Belgium. Detailed procedures are described in Tai et al. (2013).

### Neuroimaging Procedures

MRI images were obtained with a 3-T Siemens Trio Scanner at the MRISC of the University of Kentucky using a 32-channel head coil. High-resolution anatomic images (20 min 3D MPRAGE) were acquired using a rapid gradient echo acquisition sequence (acquisition matrix 256 × 256 × 176, isotropic 1 mm voxels, field of view 256 mm, repetition time 2530 ms, echo time 2.26 ms). The resting-state functional MRI (fMRI) was acquired with the following parameters: TR = 2 s; TE = 30 ms; flip angle = 76°; 39 axial slices; FOV = 224 mm × 224 mm; slice thickness = 3.5 mm; matrix = 64 × 64; bandwidth = 2056 Hz/Px. A total of 200 volumes of resting-state data were acquired. The task-based fMRI scan was acquired using a T2*-weighted gradient echo EPI sequence TR = 2 s; E = 30 ms; flip angle = 81°; 39 axial slices; FOV = 224 mm × 224 mm; slice thickness = 3.5 mm; matrix = 64 × 64; bandwidth = 2056 Hz/Px.

#### The Short-Term Memory Task

The participants performed a modified version of a visual working memory task (delayed-match-to-sample task with repeated retrieval of memory targets and distractors) that has been validated in healthy young subjects (Jiang et al., 2000). The task used two-dimensional pictures of common objects taken from Snodgrass and Vanderwart (1980). In the typical delayed match-to-sample paradigm, the subjects were first shown an item to hold in working memory at the beginning of a trial, and then determine whether a later encountered test item is a match or non-match. In the current 10-min older-adult friendly version, two sample pictures were encoded for each given trial. This reduces scanning time for older adults and increases the number of matches with balanced number of non-matches. Task-induced default network activity was calculated as a whole, with multiple cognitive components, in contrast to resting-state connectivity. Details of the event-related task are presented in Supplementary Material. Participants were trained on the memory task before the scanning session.

### Functional Connectivity Analysis of Resting State and Task

Resting-state fMRI and memory task fMRI data were preprocessed for time alignment and scanning artifact removal. For each fMRI run, the first five dummy scans (subjects were reading instructions) were discarded to eliminate typical transients at the beginning of each run. The remaining functional images were processed using SPM8.^1^ Slice timing of each functional scan was corrected to compensate for acquisition delays across slices. Motion artifacts were also estimated and corrected by realigning all functional images to the first image. The motion corrected functional images were co-registered onto the T1 structural image, then normalized to the standard MNI T1 template, and resampled into 3 mm isotropic voxels. Functional images in the MNI template space were spatially smoothed with an 8 mm full-width at half maximum (FWHM) isotropic Gaussian kernel.

Group independent component analysis (ICA) implemented in the GIFT Toolbox^2^ was applied to the smoothed resting-state fMRI images. The components that contained mPFC, PCC/Precunues, bilateral angular gyrus (AG) in the inferior parietal gyri (BA 39), and bilateral anterior temporal gyri [middle temporal Gyri (MTG), BA21, and BA20], which are known regions of the DMN, were identified and selected. For each DMN region, a region of interest (ROI) was defined to contain voxels within a sphere of 5 mm in radius centered at the voxel with the local maximum *t*-value.

Time series were extracted from all the voxels in each spherical ROI for both resting-state and task data. Residuals from nine nuisance signals, including six movement variables and three averaged signals from WM, CSF, and whole brain, were produced by regression with the time series. For resting-state data, time series were band pass filtered between 0.01 and 0.1 Hz with a finite impulse response (FIR) filter. For task functional data, each run was divided into two blocks. A total of four blocks were created from 2 runs of task fMRI. Each block contained four trials. After removing the global effects from the BOLD time series, the temporal mean was removed from each of the four blocks of time series, and the task-evoked effect was also removed by subtracting the ensemble mean of the four blocks of time series. These mean-removed time series were then used for connectivity analysis (Ding et al., 2006).

Two measures of FC, i.e., CC and TI were computed for both resting-state and task data. Although CC is the most commonly used measure for FC analysis, it only exploits the zero-lag covariance structure of the data. FMRI BOLD signals, however, are time series. A hallmark of a time series is the presence of temporal correlations at non-zero lags (Wen et al., 2012). Thus, in addition to the commonly used CC method, we also applied TI as another FC measure, which takes into account the temporal relationship beyond the zero lag (Wen et al., 2012). (see Supplementary Material for additional description and equations for the TI method).

### Statistical Analysis

We used general linear regression to identify connectivity measures significantly associated with cognition and CSF-based measures of Aβ_42_ (pg/ml) and pTau_181_ (pg/ml). The UDS *T*-score was used in the regression models rather than MMSE due to the wider range of scores. Normal distribution of these endpoints was assessed with the Shapiro–Wilk test. We modeled resting and task-based connectivity measures independently. Analyses were further stratified by measure type (CC or TI) such that four models were fit to the data for each outcome (UDS *T*-score, Aβ_42_ [pg/ml], and pTau_181_ [pg/ml]): resting state, CC connectivity; resting state, TI connectivity; task, CC connectivity; and task, TI connectivity. In each case, the initial model included all CC or TI connectivity measures relevant to resting or task, along with participant age (in years), sex (male = 0, female = 1), and years of education.

To account for multi-colinearity among the connectivity measures, as well as the high number of predictors versus subjects, we used the least absolute shrinkage and selection operator (LASSO) to identify the best subset of candidate predictors for each model (Tibshirani, 1996). The tuning parameter for each LASSO model was selected via cross-validation using PROC GLMSELECT (SAS 9.3^®^). For instance, to estimate mean Aβ_42_ using CC connectivity measures, the initial regression equation prior to application of the LASSO was as follows: Aβ_42_ = β_0_ + β_1_*age + β_2_*sex + β_3_*education + β_4_*PCC–mPFC_CC_ + β_5_* PCC–lAG_CC_ + β_6_* PCC–rAG_CC_ + β_7_* PCC–lMTG_CC_ + β_8_* PCC–rMTG_CC_ + β_9_* mPFC–lAG_CC_ + β_10_*mPFC–rAG_CC_ + β_11_*mPFC–lMTG_CC_ + β_12_*mPFC–rMTG_CC_ + β_13_*lAG–rAG_CC_ + β_14_*lAG–lMTG_CC_ + β_15_*lAG–rMTG_CC_ + β_16_*rAG–lMTG_CC_ + β_17_*rAG–rMTG_CC_ + β_18_*lMTG–rMTG_CC_. Backwards selection was then used to fit the final models on the reduced set of predictors using PROC REG (SAS 9.3^®^). LASSO reduced the number of predictors to be placed into backwards regression models from 18 to a median of 4 (range 2–9). We retained variables in the final models if they met statistical significance at 0.05. No variables were forced into the models.

## Results

### Characteristics of the Participants

All 27 participants were cognitively intact at the time of the neuroimaging and CSF biomarker study. The means and SDs of age (77), education and neuropsychological scores (MMSE, UDS *T*-scores), and their CSF biomarkers (Aβ_42_, pTau_181_, and Aβ_42_/pTau_181_) are reported in Table 1. The median of the MMSE scores was the ceiling score of 30, with interquartile range of 27–30. Mean UDS *T*-scores were over 0.5 SDs above the mean of 50 (56.8).

**Table 1:** **Characteristics of UK-ADC biomarker study participants (*n* = 27)**.

Characteristics	Summary
Age at fMRI	77.1 ± 7.4
Education	16.8 ± 2.3
Male (%)	48.2
MMSE (global cognition)	29.5 ± 0.7
UDS *T*-score (global cognition)	56.8 ± 6.9
Aβ_42_ (pg/ml)	496.0 ± 208.7
pTau_181_ (pg/ml)	65.7 ± 19.5
Aβ_42_ (pg/ml)/pTau_181_ (pg/ml)	8.1 ± 3.6

*All participants were cognitively normal at the time of biomarker collection and cognitive testing*.

Mean Aβ_42_ was 496 pg/ml for this sample. The method used for detection of Aβ_42_ level in the CSF sample established values >500 pg/ml as Aβ-normal (Sjo¨gren et al., 2001). Using this standard, 12 out of 27 participants were “high-risk” for future AD. Mean pTau_181_ was 65.7 pg/ml.

### DMN Regions of Interest

**Figure 1:**
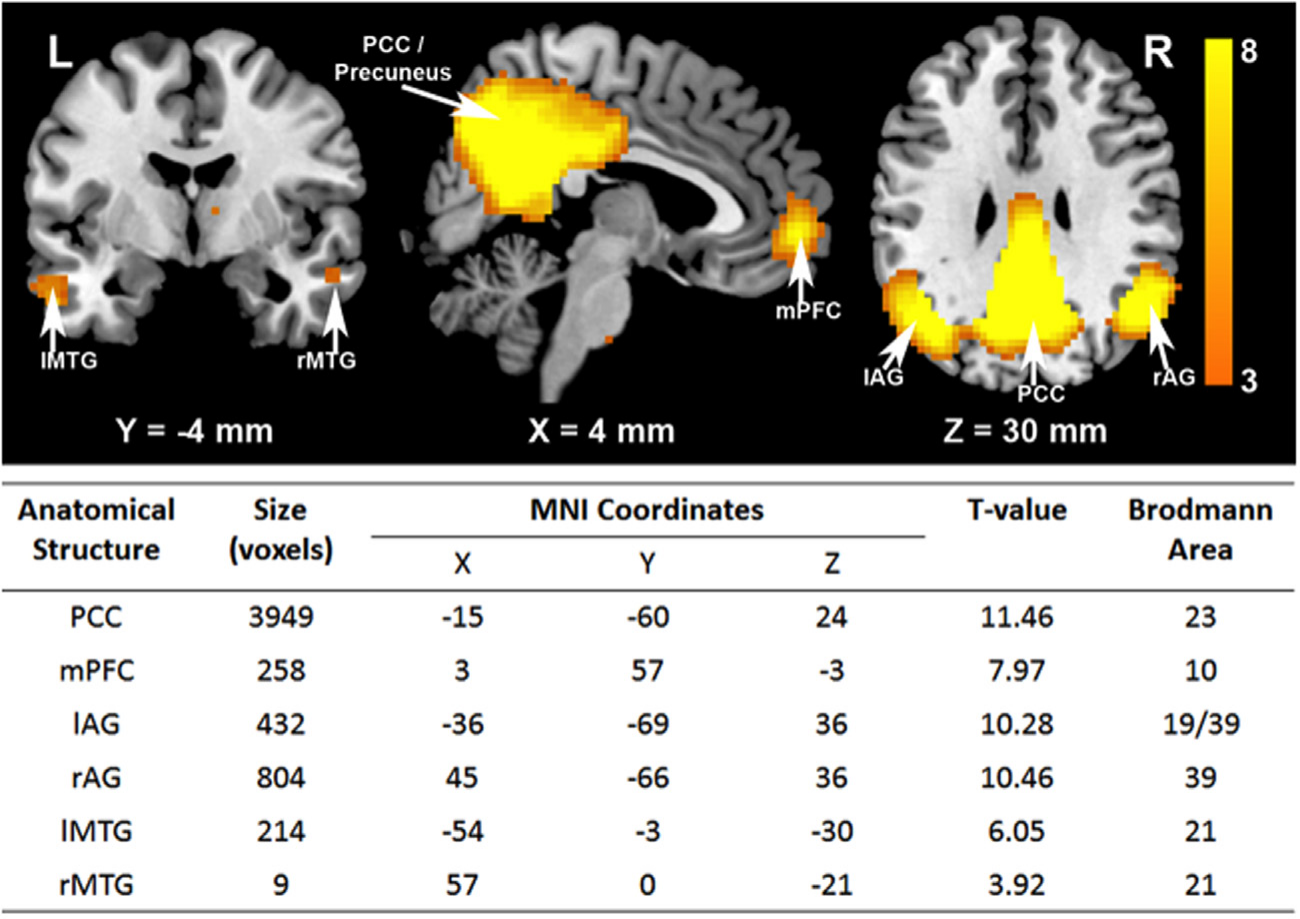
**Independent component analysis (ICA) of DMN**. Cortical regions of mPFC, PCC/Precuneus, bilateral angular gyrus (AG), and bilateral middle temporal Gyri (MTG) are activated.

Based on the ICA results shown in Figure 1, six DMN ROIs were selected in this study, including PCC/Precuneus (BA 23), mPFC (BA10), bilateral AG (BA19&39), and bilateral middle temporal gyri (BA21). Additional information on the number of voxels in each ROI and statistical significance of activation is also presented in Figure 1.

### Accuracy of the Performance during the Short-Term Memory Task

The accuracy of memory performance was examined to ensure that the participants were actually engaging on cognitive activity during task scanning. The overall accuracy for the task was about 90%. Two of the older participants did the task well but did not switch hands to indicate target match/non-match response between the two 5-min fMRI memory runs. However, for the purpose of the current study, calculated accuracy is less important than participant engagement in the task (short-term memory encoding, retrieval, and visual fixation/perception), allowing contrast with the resting state and calculation of FC over the entire task period.

### CSF Aβ_42_, Aβ_42_/pTau_181_ Ratio, Regional Brain Volume, DMN Connectivity, and Cognition

In our sample of 27 cognitively intact older adults, age was not significantly correlated with CSF Aβ_42_ (*r* = 0.05, *p* = 0.81), pTau_181_ (*r* = 0.11, *p* = 0.59) or UDS *T*-score (*r* = −0.16, *p* = 0.42) when FC was assessed using the CC method. However, age was correlated negatively with Aβ_42_ when FC was assessed by the TI method only during task (Table 3, *p* < 0.01). We included age in all regression models (Tables 2 and 3). Importantly, no significant correlations were found among levels of Aβ_42_, pTau_181_, regional brain volumes, or UDS *T-*scores. Brain volume analysis and results were reported previously in Gold et al. (2014).

**Table 2:** **Standardized beta coefficients for Aβ_42_ models, including resting-state functional connectivity measures [cross-correlation (CC) and total interdependence (TI)]**.

	A**β**_42_ (pg/ml)	
Model adj. *R*^2^	0.390	0.580
Connectivity measures	CC	TI

**Predictor**		
Age, 1 year		
Sex		
Education, 1 year		
PCC–mPFC		
PCC–lAG		
PCC–rAG		
PCC–lMTG		
PCC–rMTG		
mPFC–lAG		
mPFC–rAG	0.379^a^	0.592^b^
mPFC–lMTG		
mPFC–rMTG		
lAG–rAG		
lAG–lMTG		−1.00^b^
lAG–rMTG		
rAG–lMTG		0.656^a^
rAG–rMTG		
lMTG–rMTG	0.474^b^	0.839^c^

*The predictors are significant at the following: ^a^*p* < 0.05; ^b^*p* < 0.01; ^c^*p* < 0.0001*.

*Standardized coefficients estimate the number of SDs the dependent variable will change given a one-SD increase in the predictor variable. All predictors included in the initial models are listed in the tables; predictors with no beta coefficients were excluded from the final models. There were no significant associations between resting-state connectivity measures and UDS *T*-score or pTau_181_*.

**Table 3:** **Standardized beta coefficients for Aβ_42_ and pTau_181_ models, including task-driven fMRI functional connectivity measures [cross-correlation (CC) and total interdependence (TI)]**.

	Dependent variable			
	Aβ_42_ (pg/ml)		pTau_181_ (pg/ml)	
Model Adj. *R*^2^	0.317	0.646	0.359	0.149
Connectivity measures	CC	TI	CC	TI
**Predictor**				
Age, 1 year		**−**0.668^b^		
Sex				
Education, 1 year				
PCC–mPFC		0.430^b^		
PCC–lAG				
PCC–rAG		**−**0.526^b^		
PCC–lMTG				
PCC–rMTG	**−**0.395^a^			
mPFC–lAG				
mPFC–rAG				
mPFC–lMTG	**−**0.418^a^	−0.901^c^	**−**0.401^a^	
mPFC–rMTG				
lAG–rAG			0.591^b^	0.428^a^
lAG–lMTG				
lAG–rMTG				
rAG–lMTG		1.158^c^		
rAG–rMTG				
lMTG–rMTG	0.623^b^			

*The predictors are significant at the following: ^a^*p* < 0.05 level.; ^b^*p* < 0.01 level.; ^c^*p* < 0.0001 level*.

*Standardized coefficients estimate the number of SDs the dependent variable will change given a one-SD increase in the predictor variable. All predictors included in the initial models are listed in the tables; predictors with no beta coefficients were excluded from the final models*.

**Figure 2:**
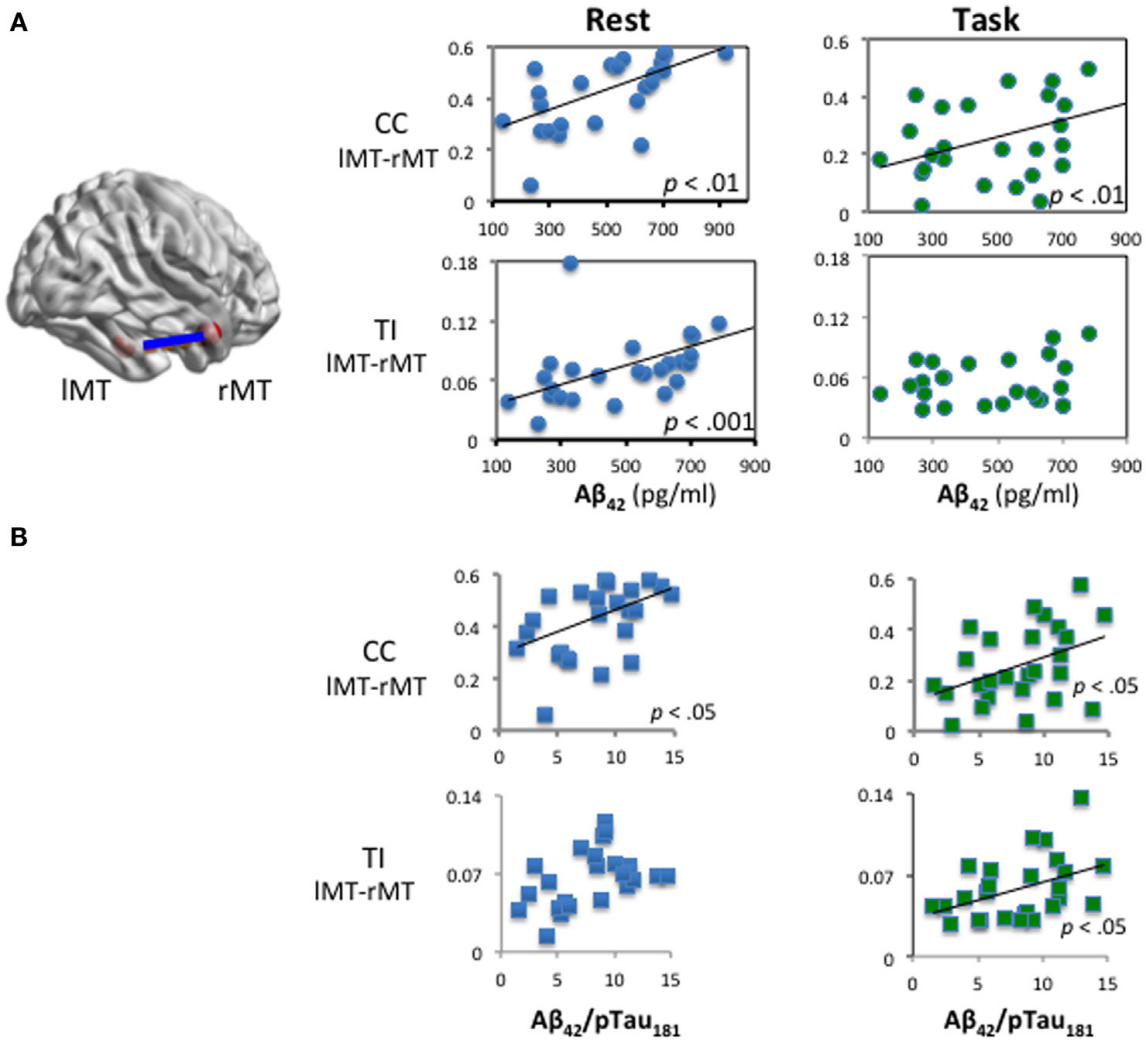
**Functional connectivity of bilateral temporal gyri and CSF Aβ_42_ and Aβ_42_/pTau_181_**. **(A)**. Aβ_42_ and bilateral anterior MTG connectivity. **(B)** Aβ_42_/pTau_181_ ratio and bilateral anterior MTG connectivity. Cognitively intact older individuals with increased bilateral MTG were associated with low risk of β-amyloid accumulations.

To avoid spurious results due to Type 1 error or inadequate statistical power (Biswal et al., 2010), we set criteria that significant correlations are reported only if they meet one of two criteria: significant results in the statistical model stronger than age-related Aβ deposits at the *p* < 0.01 level (Table 3); or, consistent results replicated in both methods (CC and TI) or during both states (resting state and task). Notably, analyses of both resting and task data revealed that FC between bilateral anterior MTG (MTG–DMN nodes, BA 21) is significantly associated with Aβ_42_ levels (*p* < 0.01; “b” or “c” in the tables) as well as Aβ/pTau_181_ ratio during resting state and task (β = 0.42 in both models, *p* < 0.05; complete model results not shown). Taking age into account, CSF Aβ_42_ level was positively correlated with bilateral connectivity of the anterior lateral temporal lobes (lMTG–rMTG) using either CC or TI analysis. Figure 2 shows that six out of eight analyses revealed that lMTG–rMTG is significantly associated with level of Aβ_42_ or Aβ_42_/pTau_181_ ratio. In other words, cognitively intact older individuals with increased bilateral MTG connectivity were associated with lower risk of β-amyloid deposits.

Another positive correlation with Aβ_42_ was found between DMN FC of left MTG and right AG in parietal lobe (Tables 2 and 3; rAG–lMTG, TI connectivity measures). That is, stronger connectivity indicates increased level of CSF Aβ_42_, implying less deposition of β-amyloid and, therefore, lower AD risk.

Brain connectivity indicating higher risk of β-amyloid deposits was also found. The strongest associations were during task: left middle temporal – mid frontal and left temporal-parietal negatively correlated with Aβ_42_ during the short-term memory task. The mPFC–lMTG connectivity during task (Table 3), and the lAG–lMTG connectivity during rest (Table 2), were negatively correlated with Aβ_42._ In other words, stronger connections were associated with lower level of CSF Aβ_42_, i.e., higher β-amyloid burden.

### CSF pTau_181_ Biomarkes and Task-Driven Functional Connectivity

**Figure 3:**
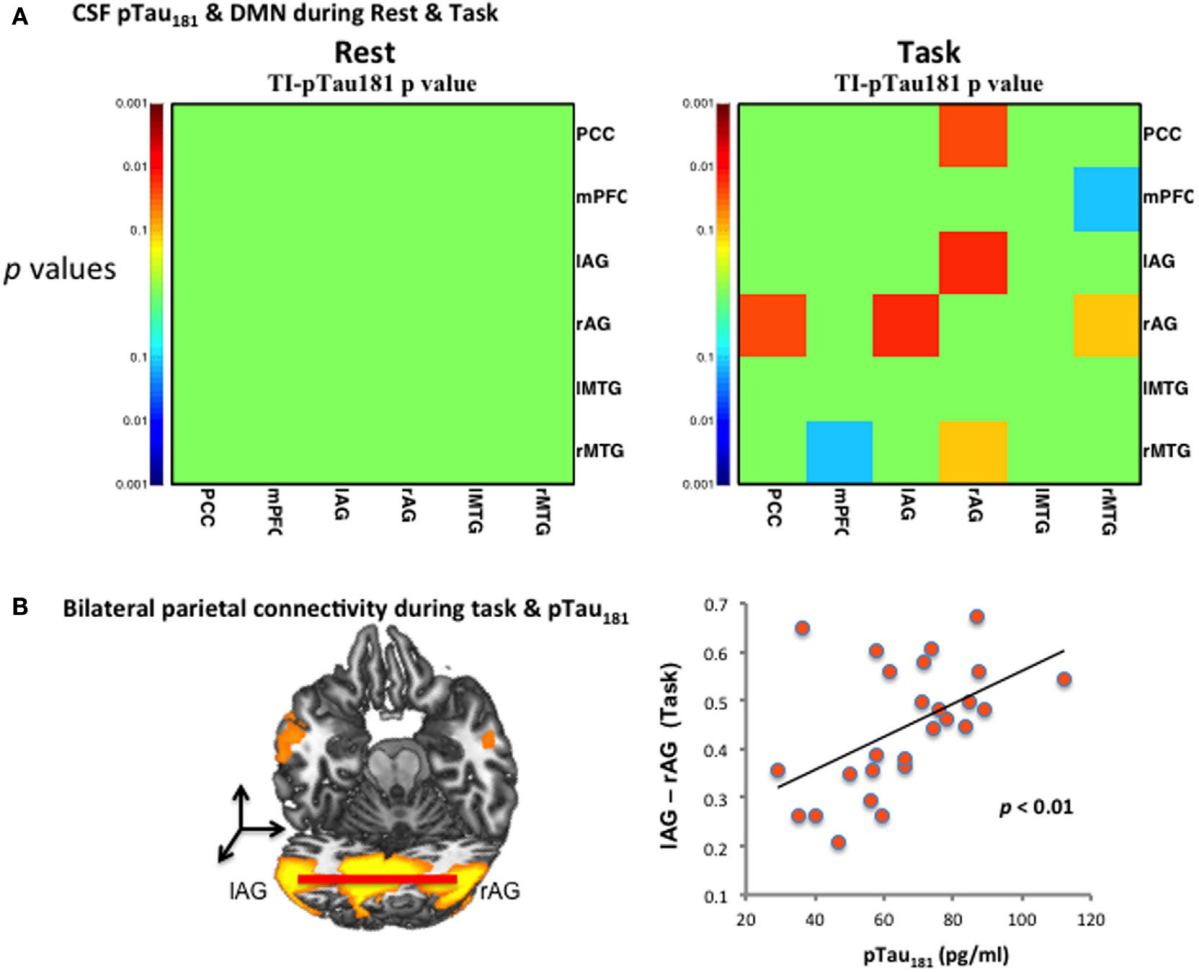
**CSF pTau_181_ level and functional connectivity during DMN**. **(A)** CSF pTau_181_ during rest and task at various significant *p*-values; the significant *p*-values (*y*-axis) of the correlation between pTau_181_ and connectivity of brain regions were found only during task, but not during resting state; **(B)** pTau_181_ and bilateral inferior parietal connectivity during task. Individuals with stronger lAG–rAG have higher level of pTau_181_, which indicates higher risk of tau-related neural degeneration.

Cerebrospinal fluid pTau_181_, but not Aβ_42_, was strongly linked to DMN FC during the short-term memory task (Table 3, lAG–rAG). Figure 3A shows the significant *p*-values (*y*-axis) of the correlation between pTau_181_ and connectivity of brain regions only during task. No significant correlations between pTau_181_ and connectivity measures were observed for the resting-state data (Figure 3A). Figure 3B shows increased pTau_181_ (higher risk for AD) is positively correlated with bilateral AG. Stronger bilateral AG connectivity was associated with higher levels of CSF pTau_181_ (pg/ml). As shown in Figure 3B, individuals with stronger FC of lAG–rAG had higher levels of pTau_181_ (Table 3).

### CSF Biomarkers, DMN Connectivity as Predictors for Global Cognition

**Figure 4:**
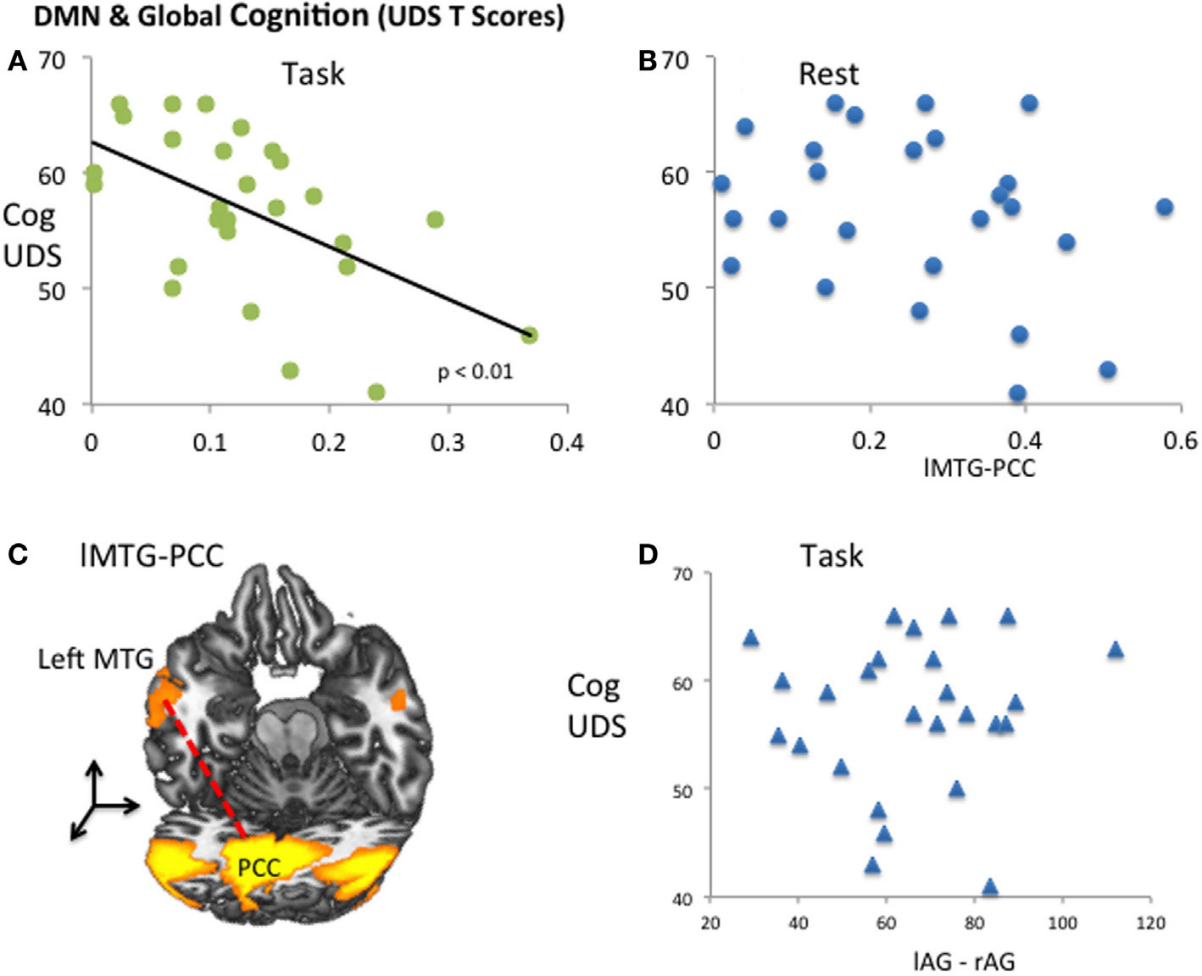
**DMN and global cognition (UDS *T*-Scores)**. **(A)** Significant correlation (*p* < 0.01) was found during task between global cognitive scores and temporal–parietal (lMT–PCC) connectivity. **(B)** During resting state, no significant correlation was found between global cognitive scores and temporal–parietal connectivity. **(C)**. Group averaged fMRI activity at the left MT and PCC/precuneus. **(D)**. Bilateral IAG, correlated with pTau, was not correlated with global cognition.

We found that global cognition, as measured by the UDS *T*-score, was not correlated with bilateral anterior temporal or bilateral parietal connectivity, nor CSF Aβ_42_ or pTau_181_ levels. UDS *T*-scores were negatively correlated with lMTG–PCC connectivity (β = −0.557, *p* < 0.01; complete model results not shown) during the memory task (Figure 4A) but not during rest (Figure 4B). Participants with stronger FC between PCC and left MTG had lower *T*-scores, suggesting a more general role of this connectivity in cognitive status. However, lMTG–PCC connectivity was not associated with CSF Aβ_42_ or pTau, putative biomarker links with AD pathology. We further tested bilateral parietal AG (Figure 4C) connectivity correlated with pTau (indicating neural degeneration), but found no correlation to *T*-scores (Figure 4D). There were no significant associations among the remaining DMN connectivity during resting-state and global cognitive function. Given the number of variables and models tested, Type 1 error is possible.

## Discussion

### Summary of the Results

**Figure 5:**
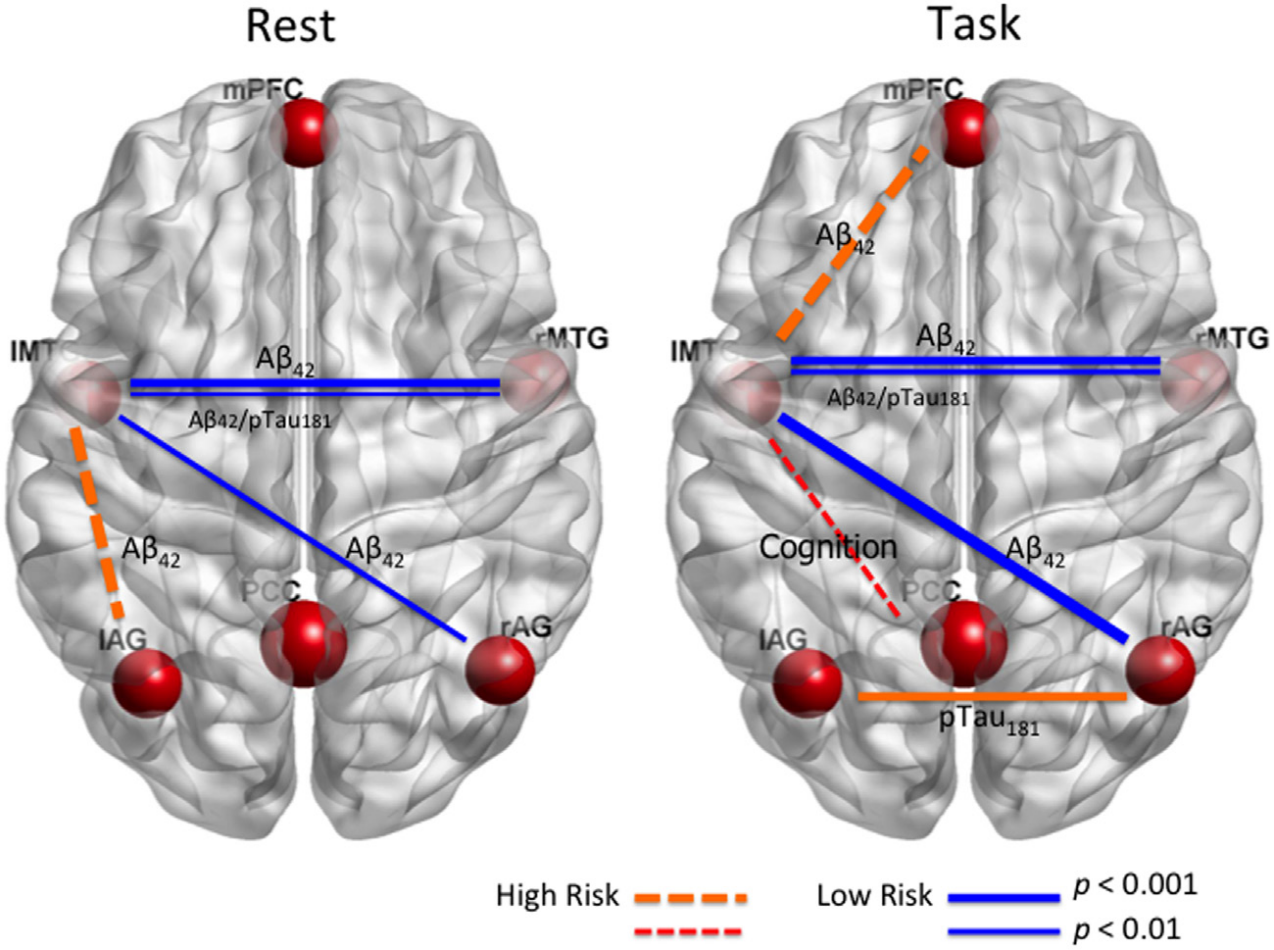
**Summary results of relations between functional brain connectivity within DMN during resting state and task, CSF biomarkers (Aβ_42_ and pTau_181_), and global cognition (*T*-scores) in cognitively normal older adults**. Blue lines indicate low AD risk, and orange/red lines indicate high AD risk. The stronger the line, the more significant the connectivity with CSF biomarker or cognition. Solid line shows that the correlation was significant in more than one analysis. Left MTG appears to be a hub for connectivity sensitive to AD risk.

We examined relationships between DMN functional brain connectivity, CSF biomarkers of AD pathology, and global cognition in cognitively intact older adults. The overall results are summarized in Figure 5. We report consistent results from six analyses which indicate that connectivity between bilateral anterior middle temporal lobe (lMTG–rMTG; BA 21) correlates positively with CSF Aβ_42_ and Aβ_42_/pTau_181_ ratio (Figure 2). This relationship was seen in the resting state and during a memory task. The left middle temporal cortex appears to be a hub of FC related to Aβ_42_ (Figure 5).

Levels of CSF pTau_181_ showed no association with resting-state connectivity but were correlated positively with bilateral parietal (lAG–rAG) connectivity during a short-term memory task. Additionally, global cognitive status negatively correlated with connectivity between left MTG and PCC only during the memory task, but this connectivity was not associated with AD biomarkers CSF Aβ_42_ or pTau. Again, only during the short-term memory task, an effect of decreasing Aβ_42_ with increasing age became apparent when DMN FC was included in the model; there was no correlation between age and Aβ_42_ otherwise.

### Neural Basis of Task-Driven Effective Connectivity and Resting-State Functional Connectivity

Our results revealed different associations between AD biomarkers and FC patterns during task performance and resting states. Previous resting-state DMN connectivity studies have reported differences in healthy older adults (Jones et al., 2011; Ferreira and Busatto, 2013), patients with AD (Vemuri et al., 2011), and patients recovering from stroke (Grefkes and Ward, 2014), suggesting that DMN connectivity is altered in specific ways in aging and certain disease states. However, the physiological nature of FC during resting state is still controversial. For instance, it is not clear whether resting-state connectivity results from anatomical connections or synchronized oscillations in brain activity (Deco et al., 2011; Eickhoff and Grefkes, 2011). Meanwhile, task-driven cortical effective connectivity may be better understood in terms of physiological basis at the synaptic level (Rehme et al., 2013).

Correlated brain activity is determined by ongoing and constantly shifting brain operations and processes engaging some networks and disengaging others (Fox et al., 2005). The DMN network activity is generally decreased during concentrated task performance and increased during the resting state, requiring the use of a relevant cognitive task as a modulator of DMN connectivity (Buckner et al., 2005, Buckner and Vincent, 2007). Since Aβ may preferentially affect component regions of the DMN and pTau is associated with synaptic degeneration, alterations in DMN connectivity may reflect multiple forms of subclinical AD pathology in both the resting and task states. Such alterations may be direct effects of the DMN itself or indirect effects mediated through synaptic interactions between DMN and other networks.

### Comparison of the Two Correlational Analyses in Relations to CSF Biomarkers

We applied two measurements, zero-lag CC and TI, to assess FC. Interestingly, the current results indicate that TI is more strongly associated with CSF Aβ_42_, while CC is more sensitive to levels of CSF pTau_181_ and global cognitive scores. Most of the previous analyses in the literature have applied the CC methods. The rationale for applying TI is that fMRI BOLD signals are time series. CC is not a time series-based measure but only exploits contemporaneous (zero lag) covariance structure of BOLD signal. The hallmark of time series is that current activity can affect the activity occurring at later times. Time series-based FC measurement, TI, takes into account the temporal dependence beyond zero lag (Wen et al., 2012). In general, the TI can capture the temporal relationship between BOLD time series, which is ignored by CC.

For the DMN during testing, both TI and CC identified positive relationships between lMTG and rMTG or mPFC and rAG with CSF Aβ_42_ levels. Compared to CC, the TI method is more sensitive to the relationship of CSF Aβ_42_ levels and other parameters (Table 3). In addition, TI revealed that increased lMTG–rAG during the resting state is associated with higher CSF Aβ_42_ level, which was further confirmed by analysis during task-driven state. In our sample of high functioning cognitively intact older adults, age alone correlated with CSF Aβ_42_ only under the task performance while using the TI method to assess the FC. This correlation result of less CSFAβ_42_ with age is consistent with known literature. Curiously, traditional CC method shows more significant correlation with levels of pTau_181_ (Tables 2 vs 3) and global cognitive score for some connections than TI (e.g., between bilateral AG).

### Bilateral Temporal Connectivity and AD Biomarker CSF Aβ_42_

Our most robust results indicate that CSF Aβ_42_ level and Aβ_42_/pTau_181_ ratio during rest or task procedures are positively correlated with bilateral middle temporal connectivity (MTG, BA 21), which was detected in all six separate analyses. Thus, it appears that increased signaling between left MTG and right temporal and parietal cortices is more possible in older adults harboring lower levels of pathology. The opposite is true between left MTG and frontal mPFC and PPC within the left hemisphere.

The anterior middle temporal cortices are critical for higher-level perception (Nakamura and Kubota, 1996), learning and memory (Arnold et al., 1994; Delacour, 1977; Lambon Ralph et al., 2009; Poettrich et al., 2009), language (Pobric et al., 2009; Schmidt and Seger, 2009), and novelty detection (Nakamura et al., 2000; Holeckova et al., 2008; McDonald et al., 2010). Additionally, the left anterior temporal cortex is considered a hub for semantic processing for object names (Shulman et al., 1997; Tsapkini et al., 2011). Typically, bilateral damage to anterior temporal cortices is required to produce a semantic deficit for object naming. Our visual working memory task includes elements of visual perception, language-assisted encoding, learning, short-term retention, and retrieval. The current findings suggest that Aβ may contribute deficits in general functions present in DMN during both resting-state and cognitive tasks.

As AD biomarkers, CSF Aβ_42_ has been validated to have high agreement with Aβ detected using positron emission tomography (PET; FDA approved F-18) imaging (Landau et al., 2013; Palmqvist et al., 2014), although there may be discrepancies in preclinical and early symptomatic AD (Blennow et al., 2015). The accuracy of both PET and CSF biomarkers in AD diagnosis is in the ~85% range (Mattsson et al., 2014). It would be interesting to validate the present finding using PET-based AD biomarkers.

### Bilateral Parietal Connectivity during Task and AD Biomarker CSF pTau_181_

Our present finding that CSF pTau correlates specifically with cortical connectivity during task performance, but not during rest, supports the idea that the presence of pTau may be associated with alterations within specific networks independently of Aβ. These alterations may be due to connections between DMN and other networks known to be affected earliest in AD, e.g., entorhinal cortex in anteromedial temporal lobe. Interestingly, pTau_181_ showed no association with resting-state DMN but positively correlated with bilateral parietal (lAG–rAG) connectivity during task, i.e., only in models that included DMN connectivity during an active memory task.

In relation to predicting AD risk in cognitively intact older adults, the current results of connectivity changes unique to task-driven state are consistent with converging evidence that cognitive impairment correlates best with the burden of neocortical neurofibrillary tangles (Nelson et al., 2012). In symptomatic AD, tau pathology is a more direct predictor of cognitive progression than Aβ (Jagust, 2013; Koch et al., 2014); together with parietal white matter lesions, tau has been shown to contribute to early development of AD (Maruyama et al., 2004; Hertze et al., 2013). Our cohort of cognitively normal older adults who later developed AD have shown synaptic loss in projections from inferior temporal lobe (Scheff et al., 2011) to PCC with progression of AD (Scheff et al., 2015). The present data support the idea that there are synergistic effects of increased tau pathology and increased level of bilateral parietal activity during cognitive tasks (Hertze et al., 2013).

### Functional Connectivity Markers and Global Cognitive Status

By contrast to CSF AD biomarkers, which showed no evidence of associations with cognitive status in the current study, FC between left temporal and parietal gyri during the short-term memory task strongly associated with overall cognitive status. Our findings support the idea that resting-state connectivity may reflect automatic and implicit cognitive activity, such as mind-wandering (Buckner and Vincent, 2007; Biswal et al., 2010), but it is not necessarily specific to higher-level cognitive tasks such as memory encoding and recall typically measured in neuropsychological tests (Broster et al., 2013). Resting-state connectivity during resting state was reported to be associated with CSF Aβ_42_ in a cohort of cognitively intact older adults (Wang et al., 2013) and in AD patients (Li et al., 2013). These results further demonstrate that FC could provide a non-invasively measured intermediate phenotype reflecting effects of the presence of Aβ and tau pathologies. This ­phenotype could serve as a biomarker of future cognitive decline and, possibly, a biomarker preceding threshold-defined abnormal levels of Aβ and tau in healthy normal older adults.

## Conclusion

In summary, we report that increased bilateral anterior middle temporal connectivity strongly correlated with higher CSF Aβ_42_ level (lower AD risk) during both resting and task states in cognitively intact older adults. Increased bilateral parietal connectivity, only during a cognitive task, is associated with higher CSF pTau_181_ (higher AD risk). FC between left anterior temporal and inferior parietal lobes during the task is associated with lower cognitive function, even though cognitive performance remained well within the normal range in all participants. These new findings suggest that brain connectivity measures during resting and task states reflect different aspects of inter-regional interactions of brain states and AD risk in cognitively normal older adults. Brain connectivity measures associated with resting and task states may, thus, serve as complementary non-invasive markers for Aβ and tau deposits and early signs of cognitive decline in healthy older adults.

## Author Contributions

YJ designed the study, contributed to data analysis, interpretations, and wrote the paper. HH conducted neuroimaging data analysis and contributed to data interpretation and writeup. EA conducted overall statistical analysis and writeup. LB contributed to experimental design, data collection, and analysis. GAJ contributed to neuroimaging and CSF data collection and analysis. FAS and RK contributed to neuropsychological data collection, statistical analysis, and writeup. AA and DP contributed to imaging data collection, analysis, and writeup. LV, BG, and PN contributed to data collection, interpretation, and writeup. CS contributed to imaging and CSF data collection, interpretation, and writeup. MD contributed to development of new computational methods, data analysis, interpretations, and writeup.

## Conflict of Interest Statement

The authors declare that the research was conducted in the absence of any commercial or financial relationships that could be construed as a potential conflict of interest. The Reviewer Subodh Kumar declares that, despite being affiliated with the same institution as the handling Editor P. Hemachandra Reddy, the review process was handled objectively.

## Footnotes

^1^http://www.fil.ion.ucl.ac.uk/spm/

^2^http://icatb.sourceforge.net/
