# Supplementary material for: Alzheimer’s Biomarkers are Correlated with Brain Connectivity in Older Adults Differentially during Resting and Task States
Source: Front Aging Neurosci. 2016 Feb 8;8:15. doi: 10.3389/fnagi.2016.00015 (PMC4744860; doi:10.3389/fnagi.2016.00015)
Supplement: Supplementary file 1 [file presentation_1.pdf]

Appendix A – The Bluegrass Short-term Memory Task

The experience of the participants in the 10-minute task was as follows. First, outside of the scanner, participants performed 3 memory trials in a practice session that was repeated up to 2 times until participants expressed comfort with the task. Next, inside the scanner, participants performed 2 runs of 8 memory trials each. At the beginning of each memory trial participants memorized two sample images with green borders (See Figure A below) and then indicated whether or not each of 12 serially presented objects matched either of the sample images via button press with the left or right hand. At the working memory retrieval stage, the subjects matched either one of the sample objects in a succession of 12 test items that contained repeated (2-4 times) matching and nonmatching items.

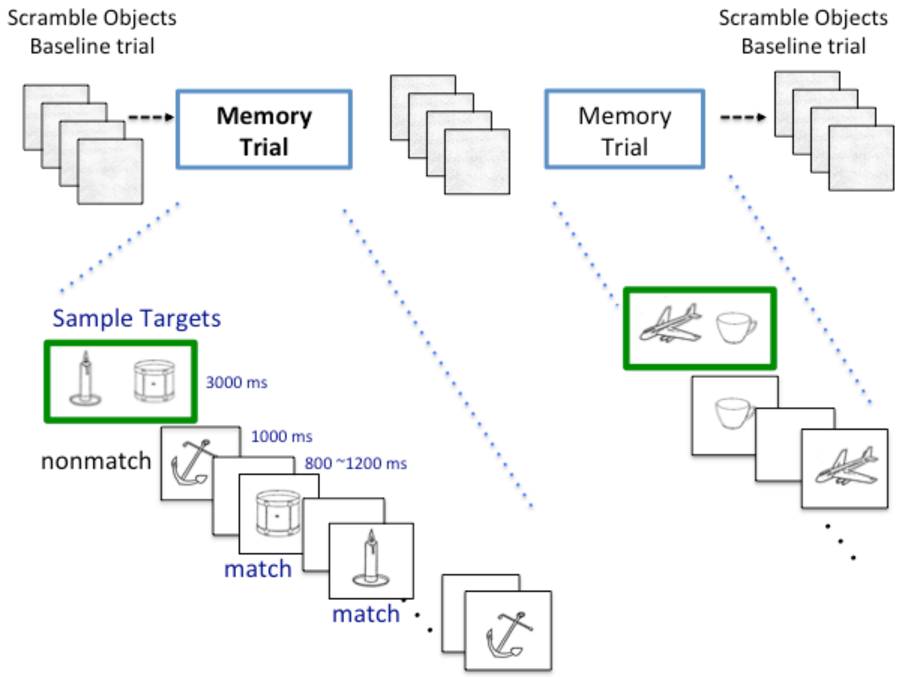

Figure A. The Bluegrass Short-term Memory Task. The task was a 10-minute version of a modified delayed match-to-sample task (Jiang et al., 2000). For each memory trial, participants memorized two sample images with green borders and then indicated whether or not each subsequently presented object (1000 ms) matched either of the sample target images.

Test pictures of matching and nonmatching objects were in a pseudo-randomized order within a trial. Each picture was presented in white color on a black background and within a rectangular area of, approximately, 8.3 by 5.8 cm (the picture subtends 10° vertically and 10° horizontally for the 32 channel head coil), and was used in exactly one trial. Each test image was triggered by the MRI scanner for 1000 ms presentation followed by a blank jittered between 800 to 1200 ms.

Between memory trials, participants passively viewed scramble images (baseline trials) from the same set of objects' images created by Fast Fourier transform (FFT) algorithms. The scrambled images in the baseline condition preserved the same spatial frequency and luminance of those object images via FFT algorithms. The hand responses were recorded via a device inside of the MRI scanner. Assignment of the response hands to indicate a match versus a nonmatch was counterbalanced within participants such that during the first run, the non-dominant hand was used to indicate a match response. The dominant hand was used during the second run to indicate a match. Each run lasted approximately 5 minutes and 30 seconds. Between the two functional MRI runs, experimenters interacted with participants to provide verbal encouragement and check for comfort with the task.

Reference:

Jiang, Y., Haxby, J. V., Martin, A., Ungerleider, L. G., Parasuraman, R. (2000). Complementary neural mechanisms for tracking items in human working memory. *Science.* 287, 643-646. doi: 10.1126/science.287.5453.643

Appendix B - Total Interdependence Measure

Two measures of functional connectivity, i.e. cross correlation (CC) and total interdependence (TI) were computed between DMN ROIs for both resting state and task data (Wen et al., 2012). Specifically, the cross correlation (zero-lag) between two time series $\boldsymbol{x}(x_1, x_2, \cdots x_n)$and $\boldsymbol{y}(y_1, y_2, \cdots y_n)$ was defined as:

$$CC_{x,y} = \left(\sum_{i=1}^{n} x_i y_i\right) \Big/ \sqrt{\left(\sum_{i=1}^{n} x_i x_i\right)\left(\sum_{i=1}^{n} y_i y_i\right)}$$

Although CC is the most commonly used measure for functional connectivity analysis, it only exploits the zero-lag covariance structure of the data. BOLD signals, however, are time series. A hallmark of a time series is the presence of temporal correlations at nonzero lags. Recent work has pointed the advantage of applying time-series based measures to resting state BOLD data (Wen et al., 2012). Here a time series based measure, total interdependence, was considered, in addition to CC. Let $\boldsymbol{X}_t = (x_t, y_t)^T$be the vector notation of the two time series from two ROIs. An autoregressive model was fit to the data, from which TI between two ROIs was derived. Let

$$\sum_{k=0}^{m} \boldsymbol{A}_k \boldsymbol{X}_{t-k} = \boldsymbol{E}_t$$

where $\boldsymbol{A}_k$ is 2×2 coefficient matrix to be estimated and $\mathbf{E}_t$ is the residual error with covariance matrix $\boldsymbol{\Sigma}$**.** The order of MVAR model is estimated by Akaike Information Criteria (AIC) (Akaike 1974). For both resting state and task state fMRI signals were found to be fitting second order MVAR model. Once the coefficient matrix $\boldsymbol{A}_k$ and $\boldsymbol{\Sigma}$ are estimated, the spectral density matrix can be defined as:

$$\boldsymbol{S}(f) = \boldsymbol{H}(f)\boldsymbol{\Sigma}\boldsymbol{H}^*(f)$$

where $\boldsymbol{H}(f) = \left(\sum_{k=0}^{m} \boldsymbol{A}_k e^{-2\pi i k f}\right)^{-1}$is the transfer function and $\boldsymbol{H}^*(f)$ is the transpose and complex conjugation of $\boldsymbol{H}(f)$. The coherence function between the two time series is defined as:

$$\boldsymbol{C}_{x,y}(f) = \frac{|S_{xy}(f)|}{|S_{xx}(f)S_{yy}(f)|^{1/2}}$$

TI was defined as,

$$TI_{x,y} = -\frac{1}{2\pi}\int_{-\pi}^{\pi} \ln\left(1 - C_{x,y}^2(f)\right) df$$

where f is the frequency and $C_{x,y}(f)$ is the ordinary coherence function. The numerical form of TI, for a given sampling frequency f_s, was computed as follows:

$$TI_{x,y} = -\frac{2}{f_s}\sum_{i=1}^{N-1} \ln\left(1 - C_{x,y}^2(i\Delta f)\right) \Delta f$$

where $\Delta f = \frac{f_s}{2(N-1)}$ is the frequency resolution and N is the number of frequency points within (0, $f_s/2$).

References:

Akaike, H. (1974). A new look at the statistical model identification. *Automatic Control, IEEE Transactions*. 19:6. doi: 10.1109/TAC.1974.1100705

Wen, X., Mo, J., Ding, M. (2012). Exploring resting-state functional connectivity with total interdependence. *Neuroimage.* 60, 1587-1595. doi: 10.1016/j.neuroimage.2012.01.079

Jiang Y, Huang H, Abner E, Broster LS, Jicha GA, Schmitt FA, Kryscio R, Andersen A, Powell D, Van Eldik L, Gold BT, Nelson PT, Smith C and Ding M (2016) Alzheimer's Biomarkers are Correlated with Brain Connectivity in Older Adults Differentially during Resting and Task States. Front. Aging Neurosci. 8:15. doi: 10.3389/fnagi.2016.00015, Supplementary materials.
